# Complete genome sequence of *Acidimicrobium ferrooxidans* type strain (ICP^T^)

**DOI:** 10.4056/sigs.1463

**Published:** 2009-07-20

**Authors:** Alicia Clum, Matt Nolan, Elke Lang, Tijana Glavina Del Rio, Hope Tice, Alex Copeland, Jan-Fang Cheng, Susan Lucas, Feng Chen, David Bruce, Lynne Goodwin, Sam Pitluck, Natalia Ivanova, Konstantinos Mavrommatis, Natalia Mikhailova, Amrita Pati, Amy Chen, Krishna Palaniappan, Markus Göker, Stefan Spring, Miriam Land, Loren Hauser, Yun-Juan Chang, Cynthia C. Jeffries, Patrick Chain, Jim Bristow, Jonathan A. Eisen, Victor Markowitz, Philip Hugenholtz, Nikos C. Kyrpides, Hans-Peter Klenk, Alla Lapidus

**Affiliations:** 1 DOE Joint Genome Institute, Walnut Creek, California, USA; 2 DSMZ - German Collection of Microorganisms and Cell Cultures GmbH, Braunschweig, Germany; 3 Los Alamos National Laboratory, Bioscience Division, Los Alamos, New Mexico USA; 4 Biological Data Management and Technology Center, Lawrence Berkeley National Laboratory, Berkeley, California, USA; 5 Oak Ridge National Laboratory, Oak Ridge, Tennessee, USA; 6 Lawrence Livermore National Laboratory, Livermore, California, USA; 7 University of California Davis Genome Center, Davis, California, USA

**Keywords:** Moderate thermophile, ferrous-iron-oxidizing, acidophile, *Acidomicrobiales*

## Abstract

*Acidimicrobium ferrooxidans* (Clark and Norris 1996) is the sole and type species of the genus, which until recently was the only genus within the actinobacterial family *Acidimicrobiaceae* and in the order *Acidomicrobiales*. Rapid oxidation of iron pyrite during autotrophic growth in the absence of an enhanced CO_2_ concentration is characteristic for *A. ferrooxidans*. Here we describe the features of this organism, together with the complete genome sequence, and annotation. This is the first complete genome sequence of the order *Acidomicrobiales*, and the 2,158,157 bp long single replicon genome with its 2038 protein coding and 54 RNA genes is part of the *** G****enomic* *** E****ncyclopedia of* *** B****acteria and* *** A****rchaea * project.

## Introduction

**Figure 1:**
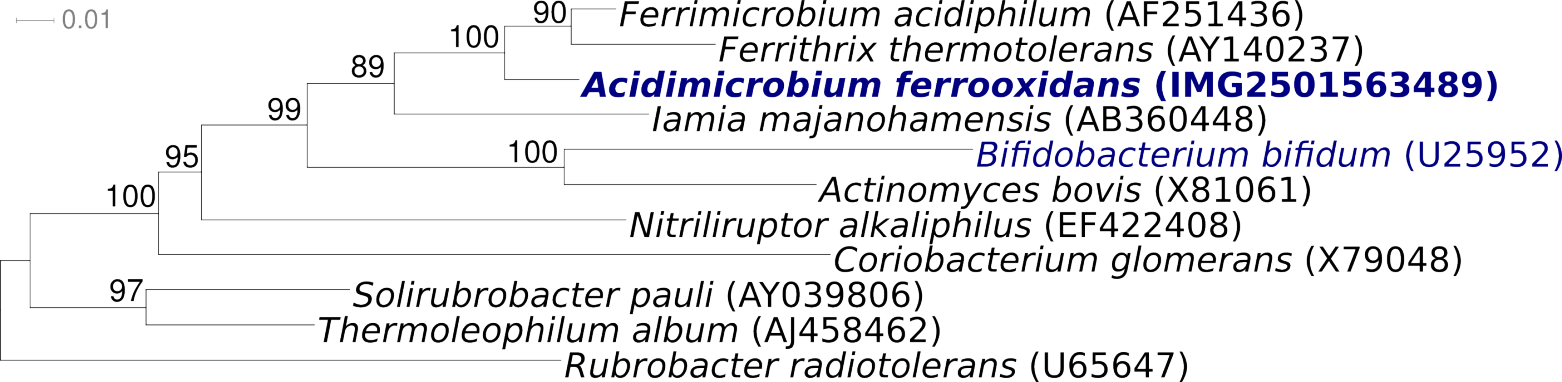
Phylogenetic tree highlighting the position of *A. ferrooxidans* strain ICP^T^ relative to all other type strains within the *Acidimicrobiales* and the type strains of all other orders within the *Actinobacteria*. The tree was inferred from 1306 aligned characters [4,5] of the 16S rRNA gene under the maximum likelihood criterion [6] and rooted with *Rubrobacteriales*. The branches are scaled in terms of the expected number of substitutions per site. Numbers above branches are support values from 1000 bootstrap replicates if larger than 60%. Lineages with type strain genome sequencing projects registered in GOLD [7] are shown in blue, published genomes in bold.

*Acidimicrobium ferrooxidans* strain ICP^T^ (DSM 10331 = NBRC 103882 = JCM 15462) is the type strain of the species, and was first isolated by Clark and Norris from hot springs in the Krísuvík geothermal area, Iceland [1,2]. For over fifteen years *A. ferrooxidans* ICP^T^ remained extremely isolated phylogenetically as the sole type strain in the actinobacterial subclass *Acidimicrobidae* [3] (Figure 1). Only at the time this manuscript was written, Kurahashi *et al.* [8] and Johnson *et al.* [9] described three novel type strains representing one novel family, *Iamiaceae* [8], and two novel genera within the *Acidimicrobiales* [9]: *Iamia majanohamensis* (isolated from sea cucumber [8]), *Ferromicrobium acidiphilum* (from a mine site in North Wales, UK [9]) and *Ferrithrix thermo-tolerans* (from Yellowstone National Park, Wyoming, USA [9]). With the exception of *I*. *majanohamensis*, all these strains live in acidic environments. Here we present a summary classification and a set of features for *A. ferrooxidans* ICP^T^ (Table 1), together with the description of the complete genomic sequencing and annotation.

**Table 1:** Classification and general features of *A. ferrooxidans* ICP^T^ based on MIGS recommendations [10]

MIGS ID	Property	Term	Evidence code^a,b^
	Current classification	Domain *Bacteria*	
		Phylum *Actinobacteria*	TAS [11]
		Class *Actinobacteria*	TAS [3]
		Order *Acidimicrobiales*	TAS [3]
		Suborder *Acidimicrobineae*	
		Family *Acidomicrobiaceae*	TAS [3]
		Genus *Acidimicrobium*	TAS [1]
		Species *Acidimicrobium ferrooxidans*	TAS [1]
		Type strain ICP	
	Gram stain	positive	TAS [1]
	Cell shape	rod shaped	TAS [1]
	Motility	motile	TAS [1]
	Sporulation	nonsporulating	TAS [1]
	Temperature range	moderate thermophile, 45-50°C	TAS [1]
	Optimum temperature	48°C	TAS [1]
	Salinity	not reported	
MIGS-22	Oxygen requirement	aerobic	TAS [1]
	Carbon source	CO_2_ (autotrophic), yeast extract (heterotrophic)	TAS [1]
	Energy source	autotrophic: oxidation of ferrous iron with oxygen as the electron acceptor; heterotrophic: yeast extract	TAS [1]
MIGS-6	Habitat	warm, acidic, iron-, sulfur-, or mineral-sulfide rich environments	TAS [1]
MIGS-15	Biotic relationship	free living	NAS
MIGS-14	Pathogenicity	none	NAS
	Biosafety level	1	TAS [12]
	Isolation	hot springs	TAS [2]
MIGS-4	Geographic location	Krísuvik geothermal area, Iceland	TAS [2]
MIGS-5	Sample collection time	before 1993	TAS [1]
MIGS-4.1 MIGS-4.2	Latitude – Longitude	63.93, -22.1	TAS [2]
MIGS-4.3	Depth	not reported	
MIGS-4.4	Altitude	not reported	

Evidence codes - IDA: Inferred from Direct Assay (first time in publication); TAS: Traceable Author Statement (i.e., a direct report exists in the literature); NAS: Non-traceable Author Statement (i.e., not directly observed for the living, isolated sample, but based on a generally accepted property for the species, or anecdotal evidence). These evidence codes are from the Gene Ontology project [13]. If the evidence code is IDA the property was directly observed for a live isolate by one of the authors or an expert mentioned in the acknowledgements.

## Classification and features

Members of the species *A. ferrooxidans* have been isolated or identified molecularly from warm, acidic, iron-, sulphur- or mineral-sulfide-rich environments. Strain TH3, isolated from a copper leaching dump [1], shares 100% 16S rRNA gene sequence identity with strain ICP^T^, whose genome sequence is reported here. The moderately thermophilic bacterium N1-45-02 (EF199986) from a spent Canadian copper sulfide heap, and strain Y00168 from a geothermal site in Yellowstone National Park [14] are the only other pure cultivated members of the species. *Acidimicrobium* species in mixed cultures used for bioleaching were frequently reported [15]. Uncultured clone sequences with significant sequence similarity (>98%) were observed by Inskeep and colleagues from several hot springs in Yellowstone National Park (e.g. AY882832, DQ179032 and others), and from hydrothermally modified volcanic soil at Mount Hood (EU419128). Screening of environmental genomic samples and surveys reported at the NCBI BLAST server indicated no closely related phylotypes (the highest observed sequence identity was 91%) that can be linked to the species or genus. Several DGGE analyses indicated the presence of members of the genus *Acidimicrobium* in metal-rich mine waters and geothermal fields around the world.

Figure 1 shows the phylogenetic neighborhood of *A. ferrooxidans* strain ICP^T^ in a 16S rRNA based tree. The sequences of the two identical copies of the 16S rRNA genes in the genome differ in 16 positions (1.1%) from the previously published 16S rRNA sequence generated from of *A. ferrooxidans* DSM 10331 (U75647). The higher sequence coverage and overall improved level of sequence quality in whole-genome sequences, as compared to ordinary gene sequences, implies that the significant difference between the genome data and the previously reported 16S rRNA gene sequence might be due to sequencing errors in the previously reported sequence data.

**Figure 2:**
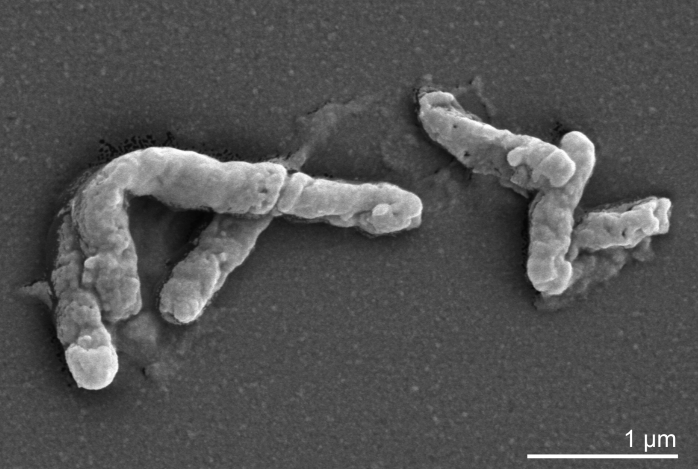
Scanning electron micrograph of *A. ferrooxidans* ICP^T^ (Manfred Rohde, Helmholtz Centre for Infection Research, Braunschweig)

Cells of strain ICP^T^ are rather small (0.4 µm × 1-1.5 µm) Gram-positive rods [1]. Optimal growth occurs at 45-50°C, pH 2, with a maximal doubling time of six hours at 48°C [1]. Cells are motile during heterotrophic growth on yeast extract. ICP^T^ forms small colonies when grown autotrophically on ferrous iron containing solid medium under air [1]. The closely related strain TH3 differs from the type strain ICP^T^ only by its tendency to grow in filaments, which has not been observed for strain ICP^T^ [1]. Strain ICP^T^ can be distinguished from members of the genus *Sulfobacillus* by its lower requirement of CO_2_ for autotrophic growth [1]. Iron oxidation by ICP^T^ cells was not influenced by supplementation of either glucose nor by increased CO_2_ concentration [1]. Thin section electron micrographs of *A. ferrooxidans* strains indicate intracellular vesicles when cells were grown on ferrous iron and yeast extract [1] (Figure 2).

### Chemotaxonomy

The murein of *A. ferrooxidans* ICP^T^ contains meso-DAP, like all other characterized type species from the *Acidomicrobineae* [8,9]. It differs from the other characterized *Acidomicrobineae* strains in MK-9(H_8_) being the predominant menaquinone, whereas *F. acidiphilum* has MK-8(H_10_) as the predominant menaquinone [9], and *I*. *majanohamensis* possesses a mixture MK-9(H_6_), MK-9(H_4_), and MK-9(H_8_) [8]. The major cellular fatty acids of strain ICP^T^ are saturated branched acids: iso- (i-) C_16:0_ (83%) and anteiso- (ai-) C_17:0_ (8%) [8], which is more similar to *F. thermotolerans* (90% i-C_16:0_) and *F. acidiphilum* (64% i-C_16:0_ and 11% i-C_14:0_) [9], than to *I. majanohamensis w*hich predominantly possesses straight chain acids (C_17:0_, C_16:0_ and C_15:0_) [8].

## Genome sequencing and annotation Genome project history

This organism was selected for sequencing on the basis of each phylogenetic position, and is part of the ***G****enomic* ***E****ncyclopedia of* ***B****acteria and* ***A****rchaea* project. The genome project is deposited in the Genome OnLine Database [7] and the complete genome sequence in GenBank (CP001631). Sequencing, finishing and annotation were performed by the DOE Joint Genome Institute (JGI). A summary of the project information is shown in Table 2.

**Table 2:** Genome sequencing project information

**MIGS ID**	**Property**	**Term**
MIGS-31	Finishing quality	Finished
MIGS-28	Libraries used	One Sanger library: 8kb pMCL200 One 454 pyrosequence standard library and one Illumina library
MIGS-29	Sequencing platforms	ABI3730, 454 GS FLX, Illumina GA
MIGS-31.2	Sequencing coverage	6.8 x Sanger; 52.9 x pyrosequence
MIGS-30	Assemblers	Newbler, Arachne
MIGS-32	Gene calling method	Prodigal
	INSDC / Genbank ID	CP001631
	Genbank Date of Release	not available
	GOLD ID	Gc01023
	Database: IMG-GEBA	2501533204
MIGS-13	Source material identifier	DSM 10331
	Project relevance	Tree of Life, GEBA

### Growth conditions and DNA isolation

*A. ferrooxidans* strain ICP^T^, DSM 10331, was grown in DSMZ medium 709 (*Acidimicrobium* Medium) at 45°C. DNA was isolated from 1-1.5 g of cell paste using Qiagen Genomic 500 DNA Kit (Qiagen, Hilden, Germany) with a modified protocol for cell lysis containing an additional 200μl lysozyme and doubled (1 hour) incubation at 37°C.

### Genome sequencing and assembly

The genome was sequenced using a combination of Sanger, 454 and Illumina sequencing platforms. All general aspects of library construction and sequencing performed at the JGI can be found at the JGI web site. 454 Pyrosequencing reads were assembled using the Newbler assembler version 1.1.02.15 (Roche). Large Newbler contigs were broken into 2356 overlapping fragments of 1000bp and entered into assembly as pseudo-reads. The sequences were assigned quality scores based on Newbler consensus q-scores with modifications to account for overlap redundancy and adjust inflated q-scores. A hybrid 454/Sanger assembly was made using the Arachne assembler. Possible mis-assemblies were corrected and gaps between contigs were closed by custom primer walks from sub-clones or PCR products. 118 Sanger finishing reads were produced. Illumina reads were used to improve the final consensus quality using an in-house developed tool (the Polisher). The error rate of the completed genome sequence is less than 1 in 100,000. Together, the combination of the Sanger and 454 sequencing platforms provided 59.7 x coverage of the genome.

### Genome annotation

Genes were identified using Prodigal [16] as part of the Oak Ridge National Laboratory genome annotation pipeline, followed by a round of manual curation using the JGI GenePRIMP pipeline [17]. The predicted CDSs were translated and used to search the National Center for Biotechnology Information (NCBI) nonredundant database, UniProt, TIGRFam, Pfam, PRIAM, KEGG, COG, and InterPro databases. Additional gene prediction analysis and functional annotation was performed within the Integrated Microbial Genomes (IMG-ER) platform [18].

## Genome properties

**Figure 3:**
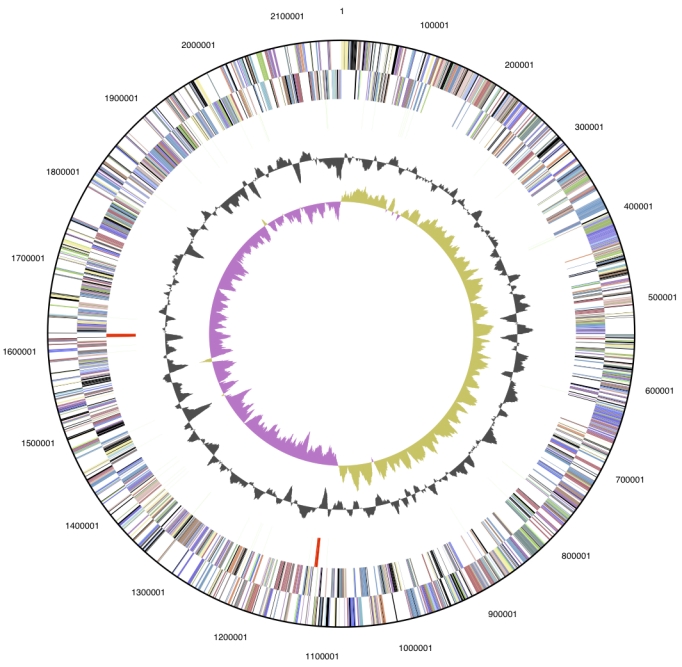
Graphical circular map of the genome. From outside to the center: Genes on forward strand (color by COG categories), Genes on reverse strand (color by COG categories), RNA genes (tRNAs green, rRNAs red, other RNAs black), GC content, GC skew.

The genome is 2,158,157 bp long and comprises one main circular chromosome with a 68.3% GC content (Table 3 and Figure 3). Of the 2092 genes predicted, 2038 were protein coding genes, and 54 RNAs. Seventy four pseudogenes were also identified. A total of 75.7% of the genes were assigned a putative function while the remaining ones were annotated as hypothetical proteins. The distribution of genes into COGs functional categories is presented in Table 4.

**Table 3:** Genome Statistics

**Attribute**	**Value**	**% of Total**
Genome size (bp)	2,158,157	100.00%
DNA Coding region (bp)	1,988,736	92.15%
DNA G+C content (bp)	1,473,791	68.29%
Number of replicons	1	
Extrachromosomal elements	0	
Total genes	2092	100.00%
RNA genes	54	2.58%
rRNA operons	2	
Protein-coding genes	2038	97.42%
Pseudo genes	74	3.54%
Genes with function prediction	1584	75.72%
Genes in paralog clusters	1969	9.37%
Genes assigned to COGs	1526	72.94%
Genes assigned Pfam domains	1603	76.63%
Genes with signal peptides	591	28.25%
Genes with transmembrane helices	436	20.84%
CRISPR repeats	2	

**Table 4:** Number of genes associated with the 21 general COG functional categories

**Code**	**Value**	**%age**	**Description**
J	134	6.6	Translation, ribosomal structure and biogenesis
A	1	0.0	RNA processing and modification
K	94	4.6	Transcription
L	119	5.8	Replication, recombination and repair
B	1	0.0	Chromatin structure and dynamics
D	24	1.2	Cell cycle control, mitosis and meiosis
Y	0	0.0	Nuclear structure
V	22	1.1	Defense mechanisms
T	73	3.6	Signal transduction mechanisms
M	84	4.1	Cell wall/membrane biogenesis
N	31	1.5	Cell motility
Z	0	0.0	Cytoskeleton
W	0	0.0	Extracellular structures
O	70	3.4	Posttranslational modification, protein turnover, chaperones
C	149	7.3	Energy production and conversion
G	87	4.3	Carbohydrate transport and metabolism
E	172	8.4	Amino acid transport and metabolism
F	54	2.6	Nucleotide transport and metabolism
H	110	5.4	Coenzyme transport and metabolism
I	86	4.2	Lipid transport and metabolism
P	60	2.9	Inorganic ion transport and metabolism
Q	34	1.7	Secondary metabolites biosynthesis, transport and catabolism
R	151	7.4	General function prediction only
S	98	4.8	Function unknown
-	512	25.1	Not in COGs
